# In Vivo Imaging of Far-red Fluorescent Proteins after DNA Electrotransfer to Muscle Tissue

**DOI:** 10.1007/s12575-009-9005-0

**Published:** 2009-04-10

**Authors:** Pernille Hojman, Jens Eriksen, Julie Gehl

**Affiliations:** 1 Centre of Inflammation and Metabolism, Department of Infectious Diseases, Rigshospitalet, University of Copenhagen, Copenhagen, Denmark; 2 Department of Oncology, 54B1, Copenhagen University Hospital Herlev, Herlev Ringvej 75, 2730, Herlev, Denmark

**Keywords:** Electroporation, Gene delivery, Whole-body imaging, Katushka, Skeletal muscle

## Abstract

DNA electrotransfer to muscle tissue yields long-term, high levels of gene expression; showing great promise for future gene therapy. We want to characterize the novel far-red fluorescent protein Katushka as a marker for gene expression using time domain fluorescence in vivo imaging. Highly efficient transgenic expression was observed after DNA electrotransfer with 100-fold increase in fluorescent intensity. The fluorescent signal peaked 1 week after transfection and returned to background level within 4 weeks. Katushka expression was not as stable as GFP expression, which was detectable for 8 weeks. Depth and 3D analysis proved that the expression was located in the target muscle. In vivo bio-imaging using the novel Katushka fluorescent protein enables excellent evaluation of the transfection efficacy, and spatial distribution, but lacks long-term stability.

## 1. Introduction

In vivo gene delivery is gaining increasing momentum for gene therapy as delivery methods become more advanced and safe. This raises the need for precise, sensitive, and easy evaluation of gene transfection efficacy. A method that has become increasingly popular is whole-body imaging of fluorescent proteins [1]. Fluorescent proteins provide unique opportunities for non-invasive labeling and tracking of transfected cells in living animals in real time [2]. Together with the development of new systems for whole-body imaging, advancements in new fluorescent proteins offer the possibility for direct visualization of gene expression in vivo.

For deep tissue imaging, the optical window for favorable light penetration is in the near-infrared wavelengths [3], which involves proteins with emission spectra in the far-red wavelengths. Most photons in the visual spectrum are absorbed by melanin and hemoglobin in animal tissue, while photons with wavelengths longer than 1,100 nm are absorbed by water. In addition, considerable scattering of the signal occurs at lower wavelengths [3].

Recently, a novel far-red fluorescent, Katushka, was characterized [4]. This protein was derived from the sea anemone *Entacmaea quadricolor*, enhanced to perform with higher brightness and exposed to site-directed mutagenesis to generate proteins with emission spectra in the far-red region. The resulting Katushka was reported to be 7- to 10-fold brighter than other far-red fluorescent proteins, e.g., HcRed and mPlum [4], and is characterized by high pH stability and photostability [4]. Few studies have so far used Katushka for in vivo bio-imaging [3,5,6], thus in this study we wanted to characterize the potential of Katushka as a marker for gene expression, including an evaluation of the kinetics and spatial distribution of the Katushka signal.

Until recent years, the preferred method for detecting fluorescence was by use of a charge-coupled device (CCD) camera [1,7]. This approach limits the degree of details one can obtain as the camera integrates light from the object, resulting in a planar picture, which is dominated by the light contribution from the first 1–2 mm of tissue. Using new technology, biological samples can be excited with sub-nanosecond laser, while emission of individual photons is detected using a rapid photon multiplier (PMT). In this way, the arrival distribution of photons as a function of time of excitation can be collected from individual points in the tissue; allowing for precise spatial and time distribution of the emitted light. One advantage of determining the fluorescence in real time is a precise determination of the decay time for the molecular quantum mechanical transition, which is responsible for light emission. This lifetime is characteristic for different fluorochromes and by filtering light emission by lifetime, one can obtain a precise measurement, where background luminescence is eliminated. The precision also allows for construction of a 3D volumetric optic tomographic map of the fluorescence.

Our choice of method for gene delivery is DNA electrotransfer. By exposing living cells to short and intense electric pulses, position-dependent changes in the transmembrane potential are induced, rendering cells accessible for cDNA entry [8,9]. There are many reports on successful in vivo DNA electrotransfer to muscle tissue as reviewed in Mir et al. [9]. We performed the transfer using a combination of one high voltage (HV) and one low voltage (LV) pulses. A pulse combination, which has proven highly efficient for muscle tissue [10,11] and causing minimal adverse effects on the muscle tissue [12,13].

Thus, using time domain optical imaging, we wanted to investigate the novel far-red fluorescent Katushka as a marker for transgenic expression with respect to intensity, lifetime, and spatial distribution, and compare it to the well-known fluorescent marker GFP.

## 2. Materials and Methods

### Animals and muscle preparation

All animal experiments were conducted in accordance with the recommendations of the European Convention for the Protection of Vertebrate Animals used for Experimentation. Experiments were performed on 7–9-week-old female C57Black/6 or NMRI mice of own breeding. Animals were maintained in a thermostated environment under a 12-h light/dark cycle and had free access to food (Altromin pellets, Spezialfutter-Werke, Germany) and water. The animals were anesthetized 10 min prior to electrotransfer or scanning by intraperitoneal injections of Hypnorm (0.4 ml/kg, Janssen Saunderton, Buckinghamshire, UK) and Dormicum (2 mg/kg, Roche, Basel, Switzerland). For ex vivo imaging, the animals were killed by cervical dislocation and intact tibialis cranialis (TC) muscles without tendons were excised and immediately scanned.

### Plasmid constructs

The plasmids, pTagFP635, encoding Katushka (Evrogen, Russia), and phGFP-S65T, encoding the green fluorescent protein (GFP) (Clontech, Palo Alto, CA, USA) both under the control of a CMV promoter were used. DNA preparations were performed using Nucleobond AX Maxiprep kits (Machery Nagel), and the concentration and quality of the plasmid preparations were controlled by spectrophotometry. Plasmids were finally dissolved in PBS at a concentration of 0.25 μg/μl unless otherwise specified.

### In vivo DNA electrotransfer

Twenty-microliter plasmid solution was injected i.m. along the fibers into the tibialis cranialis muscle using a 29G insulin syringe. Plate electrodes with 4-mm gap were fitted around the hind legs. Good contact between electrode and skin was ensured by hair removal and the use of electrode gel (Eko-gel, Egna, Italy). The electric field was applied using the Cliniporator™ (IGEA, Italy) with the following settings: a high voltage (1,000 V/cm (applied voltage = 400 V), 100 μs) pulse followed by a low voltage (100 V/cm (applied voltage = 40 V), 400 ms) pulse with a 1-s time lag between the pulses. The Cliniporator™ provided online measurement of voltage and current.

### In vivo bio-imaging

Mice were anesthetized and placed in a custom-made bed, which allowed stable and reproducible imaging of the legs. In vivo scanning was performed using the Optix MX-2 Optical Molecular Image System (Advanced Research Technologies, Montreal, Canada), which uses time domain optical imaging. In time domain optical imaging, short pulses of light driven by pulsed laser diodes are used to illuminate the organism under study and excite fluorescent molecules. Time-of-flight distribution enables depth and concentration to be uncoupled and fluorescence lifetime to be determined. For Katushka, excitation was performed with a 635-nm (LDH-P-635) pulsing laser and emission was detected with a 650 long pass filter, while excitation of GFP was performed with a 470-nm pulsing laser and emission was detected through a 525-nm band pass filter. The scan was performed over a Cartesian grid in prioritized raster fashion, and each scan took on average 5 min.

### Data analysis

Data analysis was performed using the Optiview 2.2 software (ART Advanced Research Technologies Inc., Canada) supplied with the ART Optix bioimager. The software is used for background subtraction, lifetime analysis of the fluorochromes, depth and concentration analysis, and generation of 3D images.

The temporal dispersion of fluorescent photons is measured by time-correlated single photon counting (TCSPC) after excitation of a fluorophore by laser pulse. Analysis of this temporal dispersion curve—the fluorescent temporal point spread function (TPSF)—is used to obtain information of the in vivo fluorophore depth, concentration, and lifetime. Based on the Optix machine settings, the user will be able measure temporal and spatial distribution of fluorophores in regions in tissue: the light intensity is measured as a function of arrival time in nanoseconds, where the signal from deeper tissues arrives later allowing the estimation of relative concentration difference.

## 3. Results

### 3.1. Efficiency of Katushka expression in muscles over time

**Figure 1:**
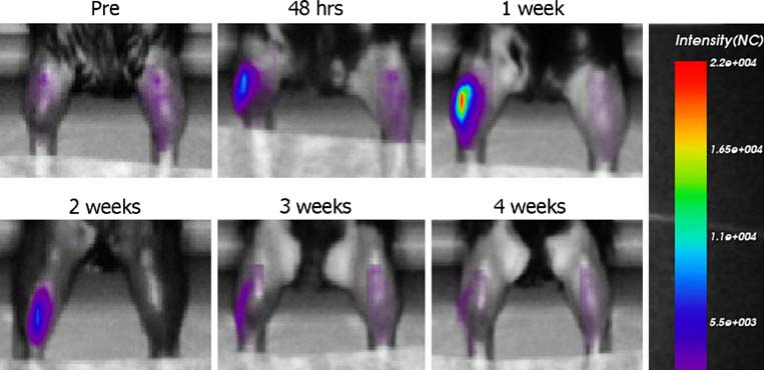
**Time course of the intensity of Katushka expression in muscles after DNA electrotransfer**. The left leg was transfected, while the right leg served as untreated control. The picture series was taken of the same mouse, but is representative of seven mice.

**Figure 2:**
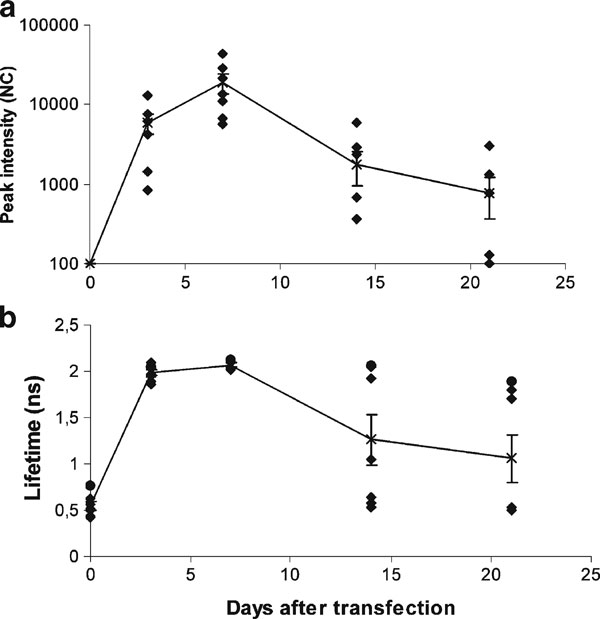
**Time course of a Katushka intensity (mean ± SD) and b Katushka lifetime (mean ± SD) in a scanning series of seven mice following DNA electrotransfer of 5 μg Katushka plasmid**.

**Figure 3:**
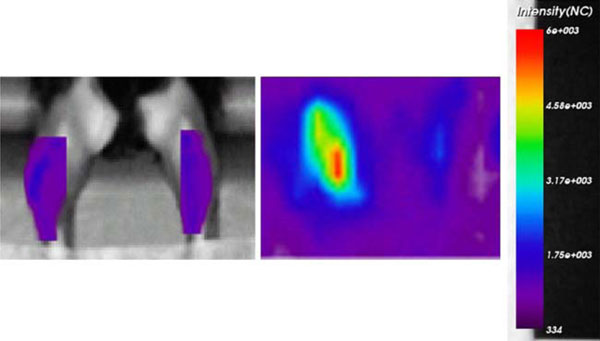
**Four weeks after DNA electrotransfer, the muscles were scanned in vivo (*left image*), and then excised and scanned ex vivo with the same settings (*right image*)**.

Electrotransfer of 5 μg of pTurboFP635 ('Katushka') plasmid resulted in a large increase in the in vivo fluorescent intensity (mean peak value = 18,695 ± 5,242 NC, *n* = 8) from the transfected muscle (Figure 1). The Katushka intensity peaked 1 week after electrotransfer, where after it leveled off and returned to background level within 4 weeks (Figure 2). To examine the sensitivity of the in vivo analysis compared with ex vivo scans, the muscles were excised at 4 weeks and scanned. Even though Katushka expression could not be detected in vivo, residual Katushka expression was present in muscles when scanned ex vivo (Figure 3).

To determine the minimum dose of Katushka plasmid needed to give detectable fluorescent intensity, we decreased the amount of pTurboFP635 to 0.5 and 1 μg, respectively. Electrotransfer with 1.0 μg of plasmid resulted in detectable fluorescent signal with an intensity of 1,090 NC, proving that as little as 1.0 μg of Katushka plasmid is detectable by in vivo imaging (data not shown).

### 3.2. Lifetime analysis of Katushka expression

**Figure 4:**
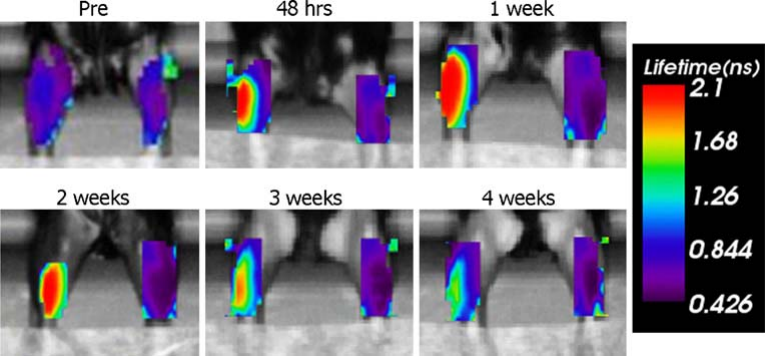
**Time course of the lifetime of Katushka expression, showing the same muscles as in (Figure 1)**.

After excitation, fluorescent proteins are characterized by a specific decay time, known as lifetime. Determination of the lifetime enables recognition of a specific protein by time domain analysis. Lifetime analysis of the transgenic Katushka signal obtained within the first 2 weeks after DNA electrotransfer showed a lifetime of 2.1 ns. This corresponds to the expected lifetime of Katushka (Figure 4). In line with the decrease in fluorescent intensity, the lifetime also decreased at 4 weeks after DNA electrotransfer (Figure 2). The temporal point spread function (TPSF) at 4 weeks showed a dual display, indicating that a real yet weak Katushka signal was mixed with the background signal (data not shown).

### 3.3. Comparison of Katushka versus GFP expression

**Figure 5:**
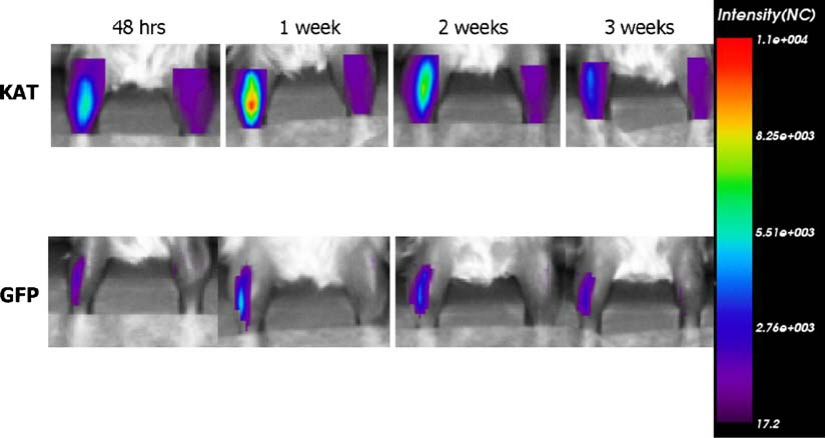
**Comparison of Katushka and GFP expression in muscles after DNA electrotransfer**. Intensity of Katushka or GFP followed over time and the color scale is set to the same range for both Katushka and GFP. The left leg was transfected, while the right leg served as control. The pictures are representative of four mice for each gene.

To compare the efficacy of Katushka with GFP, which has been used extensively for imaging, a scanning series comparing the two was performed (Figure 5). Again, the fluorescent intensity of Katushka peaked at 1 week after DNA electrotransfer and returned to background level within 4 weeks. The same pattern of GFP intensity was present with peak intensity obtained 1 week after DNA electrotransfer. GFP, however, did not show the same degree of decrease in fluorescent intensity and the signal remained detectable for at least 8 weeks. Looking at the lifetime analyses, Katushka lifetime decreased at 3 weeks after treatment, while GFP lifetime remained stable for at least 6 weeks (data not shown).

### 3.4. 3D distribution of Katushka expression

**Figure 6:**
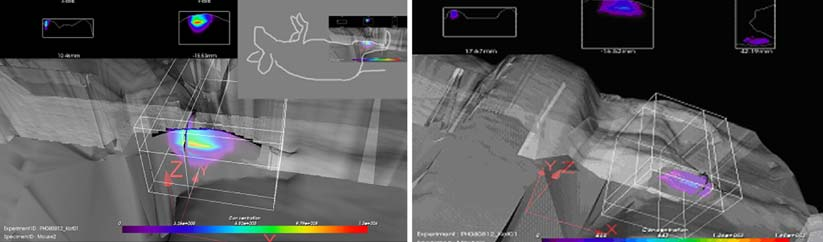
**3D analysis of Katushka (*left panel*) and GFP (*right panel*) expression in muscles 1 week after DNA electrotransfer**. The 3D analysis allows us to determine the spatial distribution of the Katushka expression, which in this case coincides with the location of the tibialis cranialis muscle.

The time-of-flight imaging acquisition enabled us to determine the spatial distribution of the fluorescent signal. Through 3D analysis (Figure 6) we determined the spatial location of both the Katushka and GFP signal in muscles 1 week after DNA electrotransfer. For Katushka, the fluorescent signal was located 0.1 mm from the top of the leg, reaching 5.6 mm down. From the side of the leg, the fluorescent signal ranged from 1 mm under the skin to 5 mm inside the leg. The longitude of the fluorescent signal was 5 mm. GFP expression was approximately located in the same area. This volume is equivalent to the location of the tibialis cranialis muscle, which was the intended target for the DNA electrotransfer.

## 4. Discussion

Bio-imaging shows great advantages for detection of gene expression in vivo [1,14]. In this study, we report highly efficient Katushka expression in muscles after DNA electrotransfer with little background expression. As little as 1.0 μg Katushka plasmid is sufficient for in vivo detection. Due to the favorable light penetration in the infrared region, precise determination of the spatial expression in the tissue was possible, and we found the Katushka signal to be located in the region corresponding to the tibialis cranialis muscle. Compared to GFP, the Katushka signal had significantly higher peak intensity in the first weeks; however, the signal was of shorter duration and in vivo detection was lost after 4 weeks.

DNA electrotransfer is a highly efficient method for in vivo gene delivery with high level and long-term expression of the transferred genes [15,16]. For in vivo imaging of muscular transgenic expression, penetrating and stable signals are needed. In vivo imaging with Katushka offered high sensitivity and precision of the signal, but the signal decayed over a few weeks. This shows that even though the brightness of Katushka is superior to other far-red fluorescent proteins [4], Katushka is not the optimal marker for long-term expression as is obtained after DNA electrotransfer to muscle tissue. Katushka may prove useful as a marker of weak gene expression due to the superior brightness. In this study, the intensity of Katushka was 3-fold higher than GFP at 1 week after DNA electrotransfer.

A particular advantage of time domain optical imaging is the possibility to determine the spatial distribution of the fluorescent signal. We found that both Katushka and GFP expression was good tools in 3D analysis as little background and divergence of the signal occurred. Based on the geometric positions generated by the 3D analysis, we were able to give an estimate of the location of the fluorescent signal. In the transfected legs, the fluorescent signal corresponded to the location of tibialis cranialis muscle, which was the intended target of our transfections. Since we were imaging tissues, which were only 5 mm in depth, we did not find any significant differences between the two fluorochromes regarding the estimation of the spatial distribution. Far-red fluorescent proteins should in theory be superior in deep tissue imaging; however, in our case, the tibialis cranialis muscle was not located deep enough for this effect to be important.

The initial study describing Katushka proved it highly photostable with fast recovery after bleaching [4]. Thus, the weekly scans, which we performed, should not be bleaching the signal to a degree that could explain the decrease in intensity, which we observe in our experiments. Katushka has a fast maturation time (20 min); however, the half-life in vivo is unknown. Our studies showed that Katushka has a lower stability in vivo than GFP. Katushka is a newly described fluorescent molecule and few studies on the biochemical properties of Katushka exists [4], thus whether the low stability is due to intrinsic protein features or whether Katushka might be immunogenic in vivo remains to be investigated.

In conclusion, time domain optical imaging with intensity, lifetime, and 3D analyses ensured extensive characterization of Katushka expression; proving that Katushka offered excellent evaluation of the transfection efficacy with a signal much brighter than GFP. The maintenance of the fluorescent signal was, however, lost within a few weeks, showing that Katushka is not a good candidate for long-term detection of in vivo transgenic expression. Both Katushka and GFP were good markers for the spatial distribution of gene expression in the tibialis cranialis muscle.
